# Growth of Triple Negative and Progesterone Positive Breast Cancer Causes Oxidative Stress and Down-Regulates Neuroprotective Transcription Factor NPAS4 and NPAS4-Regulated Genes in Hippocampal Tissues of TumorGraft Mice—an Aging Connection

**DOI:** 10.3389/fgene.2018.00058

**Published:** 2018-03-05

**Authors:** Anna Kovalchuk, Yaroslav Ilnytskyy, Rocio Rodriguez-Juarez, Amanda Katz, David Sidransky, Bryan Kolb, Olga Kovalchuk

**Affiliations:** ^1^Department of Neuroscience, University of Lethbridge, Lethbridge, AB, Canada; ^2^Leaders in Medicine Program, Cumming School of Medicine, University of Calgary, Calgary, AB, Canada; ^3^Department of Biological Sciences, University of Lethbridge, Lethbridge, AB, Canada; ^4^Department of Oncology, Champions Oncology, Baltimore, MD, United States; ^5^Department of Otolaryngology and Oncology, Johns Hopkins University, Baltimore, MD, United States

**Keywords:** non-CNS cancer, tumor brain, brain ageing, gene expression, animal models

## Abstract

While the refinement of existing and the development of new chemotherapeutic regimens has significantly improved cancer treatment outcomes and patient survival, chemotherapy still causes many persistent side effects. Central nervous system (CNS) toxicity is of particular concern, as cancer patients experience significant deficits in memory, learning, cognition, and decision-making. These chemotherapy-induced cognitive changes are termed chemo brain, and manifest in more than half of cancer survivors. Moreover, recent studies have emerged suggesting that neurocognitive deficits manifest prior to cancer diagnosis and treatment, and thus may be associated with tumor presence, a phenomenon recently termed “tumor brain.” To dissect the molecular mechanisms of tumor brain, we used TumorGraft^TM^ models, wherein part of a patient's tumor is grafted into immune-deficient mice. Here, we analyzed molecular changes in the hippocampal tissues of mice carrying triple negative (TNBC) or progesterone receptor positive (PR+BC) xenografts. TNBC growth led to increased oxidative damage, as detected by elevated levels of 4-hydroxy-2-nonenal, a product of lipid peroxidation. Furthermore, the growth of TNBC and PR+BC tumors altered global gene expression in the murine hippocampus and affected multiple pathways implicated in PI3K-Akt and MAPK signaling, as well as other pathways crucial for the proper functioning of hippocampal neurons. TNBC and PR+BC tumor growth also led to a significant decrease in the levels of neuronal transcription factor NPAS4, a regulator that governs the expression of brain-derived neurotrophic factor (BDNF), and several other key brain neurotrophic factors and pro-survival molecules. The decreased expression of ERK1/2, NPAS4, and BDNF are also seen in neurodegenerative conditions and aging, and may constitute an important tumor brain mechanism.

## Introduction

The development of new chemotherapeutic agents and amelioration of existing protocols have significantly increased patient survival and improved treatment outcomes. Nevertheless, chemotherapy still has many long-term side effects that negatively influence the quality of life of cancer patients. Among these side effects, manifestations of central nervous system (CNS) toxicity are of particular concern (Ahles et al., 2012; Soffietti et al., 2014). Chemotherapy causes significant declines in processing and long-term memory, learning, and cognition. It interferes with sleep and decision-making and, as noted by many patients, with the very way they think. These effects are widespread across all cancer patients, but are most pronounced and were first noted by breast cancer survivors, manifesting in up to 75% of all breast cancer cases. It was breast cancer patients who first coined the term “chemo brain” to explain their condition; the term is now widely used to refer to chemotherapy-induced cognitive changes (Wefel and Schagen, 2012).

Several studies that used both pre- and post-treatment patient cohorts revealed that in 30% of patients, neurocognitive deficits manifested prior to chemotherapy. Even though these studies are scarce, they suggest that chemo brain has to be extended to include cancer-induced cognitive impairments, a concept that we have recently termed “tumor brain.” Tumor brain remains much less investigated than chemo brain. Meanwhile, chemo brain has recently gained widespread attention and has been extensively analyzed using animal models where animals were exposed to various chemotherapy agents.

The mechanisms behind chemo brain include increased oxidative stress, altered levels of neuronal proliferation and apoptosis, inhibition of neuronal differentiation, disruption of hippocampal neurogenesis, increased inflammation, disruption of the blood-brain barrier, alterations in brain blood flow, and changes in metabolism (Han et al., 2008; Joshi et al., 2010; Lyons et al., 2011; Raffa, 2011; Seigers and Fardell, 2011; Christie et al., 2012; Briones and Woods, 2014; Briones et al., 2015). Chemo brain is epigenetically regulated and linked to aberrant histone modification levels. In our recent study, we showed that exposure to mitomycin C and cyclophosphamide alters DNA methylation and global gene expression, and causes oxidative DNA damage in the prefrontal cortex and hippocampus of exposed mice.

To date, the vast majority of chemo brain studies have used animals without tumors, and the mechanisms of tumor brain are therefore under-investigated. In another set of studies, we used TumorGraft animal models that are used in precision oncology approaches to analyze the mechanisms behind tumor brain and chemo brain in tumor-bearing chemotherapy treated and untreated mice. We found that the presence of a tumor caused pronounced changes in the levels of gene and small RNA expression, global DNA methylation and hydroxymethylation, as well as in oxidative DNA damage and the levels of several neurotrophic factors (submitted). Tumor presence played a governing role in the framework of the observed effects, while chemotherapy treatments further exacerbated tumor-induced changes. Within the scope of that study, we observed pronounced molecular effects of extracranial malignant tumor growth on the prefrontal cortex of non-CNS tumor-bearing animals. While our studies looked at the prefrontal cortex, in the past, the majority of chemo brain analysis has focused on the hippocampus. However, while the effects of chemotherapy on the hippocampus have been demonstrated, changes caused by non-CNS tumor growth have never been analyzed in the hippocampal domain.

Here, we analyzed molecular changes in the hippocampal tissues of TNBC and PR+BC bearing mice. This is the first study to show that the growth of TNBC and PR+BC tumors alters global gene expression in the murine hippocampus and affects multiple pathways implicated in PI3K-Akt and MAPK signaling, as well as other pathways that are crucial for the proper functioning of hippocampal neurons. We also noted that tumor growth led to downregulation of NPAS4, BDNF, and several other neurotrophic factors.

## Results

### Impact of non-central nervous system (CNS) tumor growth on the levels of 4-hydroxynonenal in the murine hippocampus

**Figure 1:**
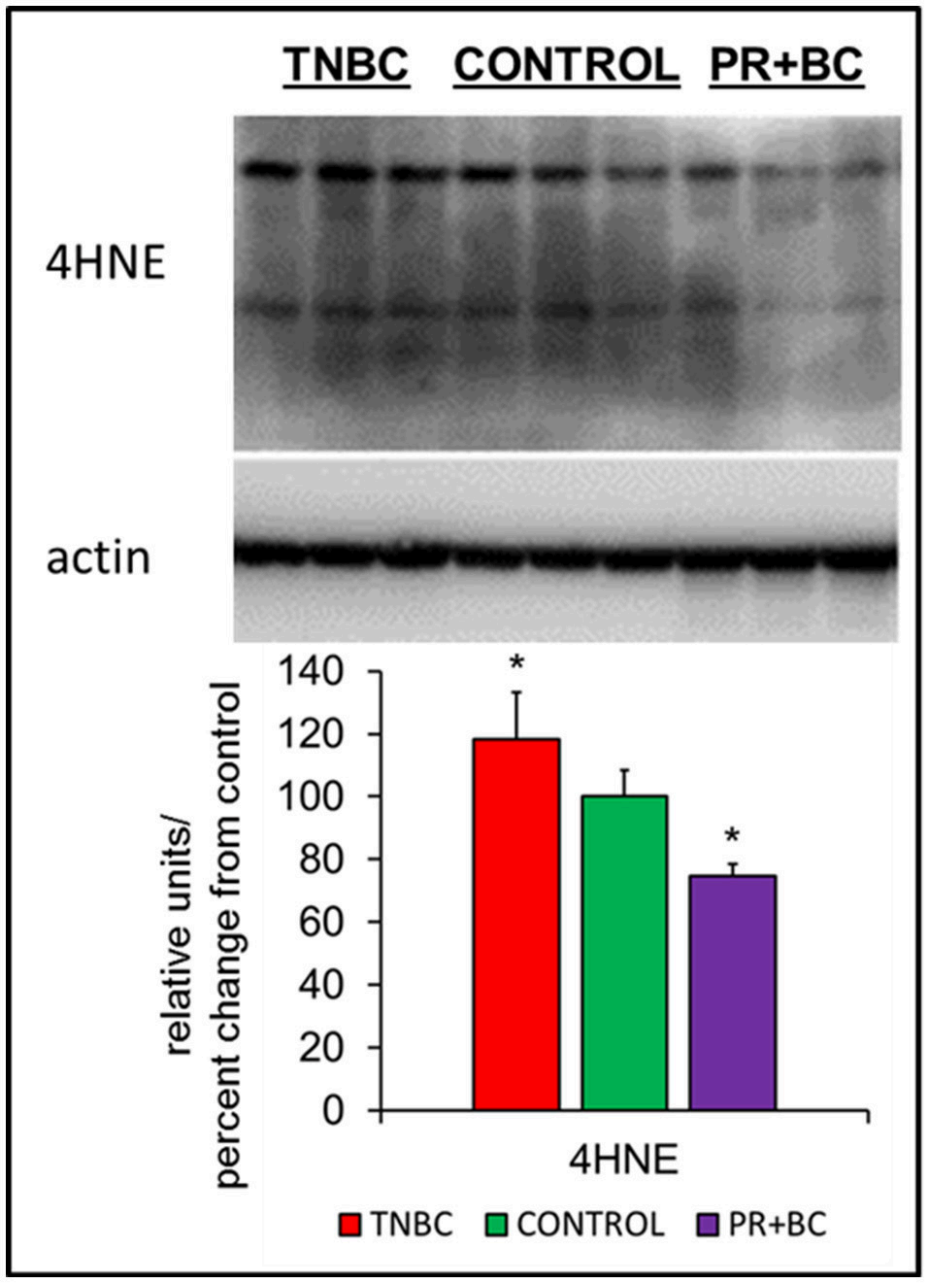
Levels of 4-hydroxy-2-nonenal (4-HNE) in the hippocampal tissues of control and TNBC and PR+BC TumorGraft mice. Western immunoblotting analysis of the levels of 4-HNE; data are shown as relative units/percent change from control; ^*^p < 0.05.

Oxidative stress is a feature of cancer, and has been implicated in chemo brain and in numerous other neurological diseases and conditions. In cells and tissues, oxidative stress leads to the generation of several lipid peroxidation end-products, one of which is 4-hydroxynonenal (4-HNE), a highly reactive aldehyde produced from the peroxidation of omega-6-polyunsaturated fatty acids. Levels of 4-HNE are biomarkers of oxidative stress. To analyze whether or not growth of triple-negative breast cancer (TNBC) or progesterone receptor-positive breast cancer (PR+BC) patient-derived xenografts (PDXs) causes oxidative stress, we evaluated the levels of 4-HNE in the hippocampal tissues of tumor-bearing mice (Vila et al., 2008; Zheng et al., 2014). Our analysis revealed that growth of malignant TNBC tumors significantly (*p* < 0.05) upregulated levels of 4-HNE in the hippocampus of tumor-bearing mice (Figure 1). On the contrary, growth of PR+BC tumors resulted in decreased levels of 4-HNE.

### Effect of triple negative and progesterone positive breast cancer growth on global gene expression in the hippocampal tissues of tumor-bearing mice

Profiling global gene expression reveals all molecular changes, both mutational and non-mutational; hence, global transcriptome analysis is the best method for understanding the entirety of molecular and cellular processes. Analyzing global gene expression provides a full picture of normal tissue development and functioning, as well as the underlying causes of diseases and conditions. To explore the effects of TNBC and PR+BC tumors on the murine brain, we used the Illumina next-generation sequencing platform to carry out a global gene expression analysis of the hippocampal tissues of PDX mice. Global gene expression profiling revealed pronounced changes in the hippocampal tissues of TNBC and PR+BC animals. Principal component analysis based on the entire gene expression dataset showed clustering of groups and differences between transcriptome profiles in the hippocampal tissues of control and TNBC and PR+BC tumor-bearing mice (Figure S1).

**Figure 2:**
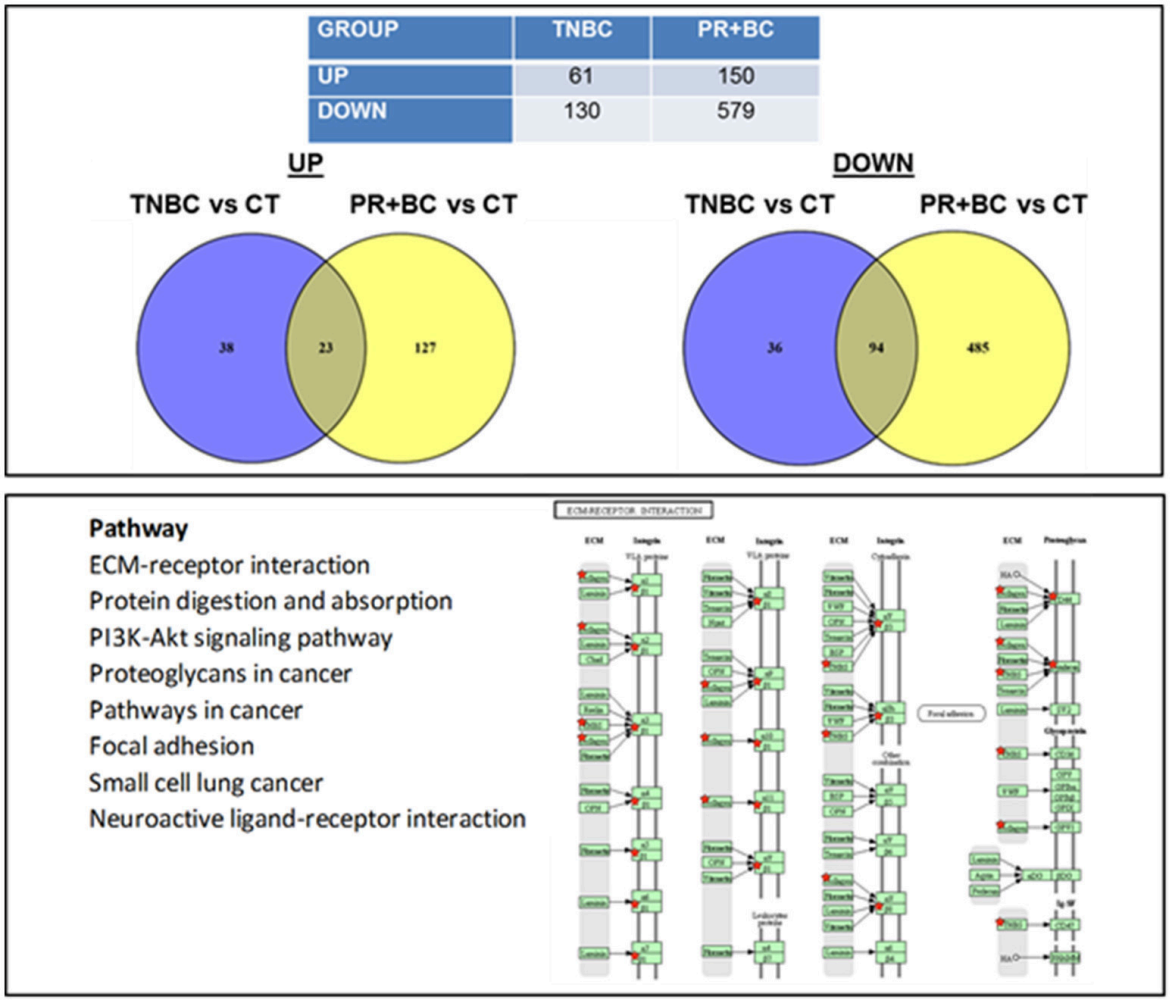
Next generation sequencing-based analysis of gene expression in the hippocampal tissues of control and TNBC and PR+BC TumorGraft mice. Table and Venn diagrams show numbers of genes that were significantly different between TNBC and PR+BC mice, as compared to controls. Below is a list of molecular pathways that were commonly down-regulated in the hippocampal tissues of TNBC and PR+BC mice. Pathview/KEGG analysis was used to determine differentially affected pathways. Figure displays the KEGG ECM-Receptor Interaction pathway.

We noted that in the hippocampal tissues of TNBC animals, 61 genes were upregulated and 130 genes were downregulated, as compared to control mice. In PR+BC animals, 150 genes were upregulated and 579 were downregulated (Figure 2). Of those, 23 genes were commonly upregulated and 94 genes were downregulated in the hippocampal tissues of both TNBC and PR+BC animals (the adjusted *p* < 0.10; the log fold change was 0.58). Commonly changed genes, both up- and downregulated, were mapped to KEGG biological pathways using the DAVID Bioinformatics and Paintomics platform. Commonly deregulated pathways included the PI3K-Akt signaling pathway, Protein digestion and absorption, ECM-receptor interactions, and Neuroactive ligand-receptor interactions (see Figure S2).

**Figure 3:**
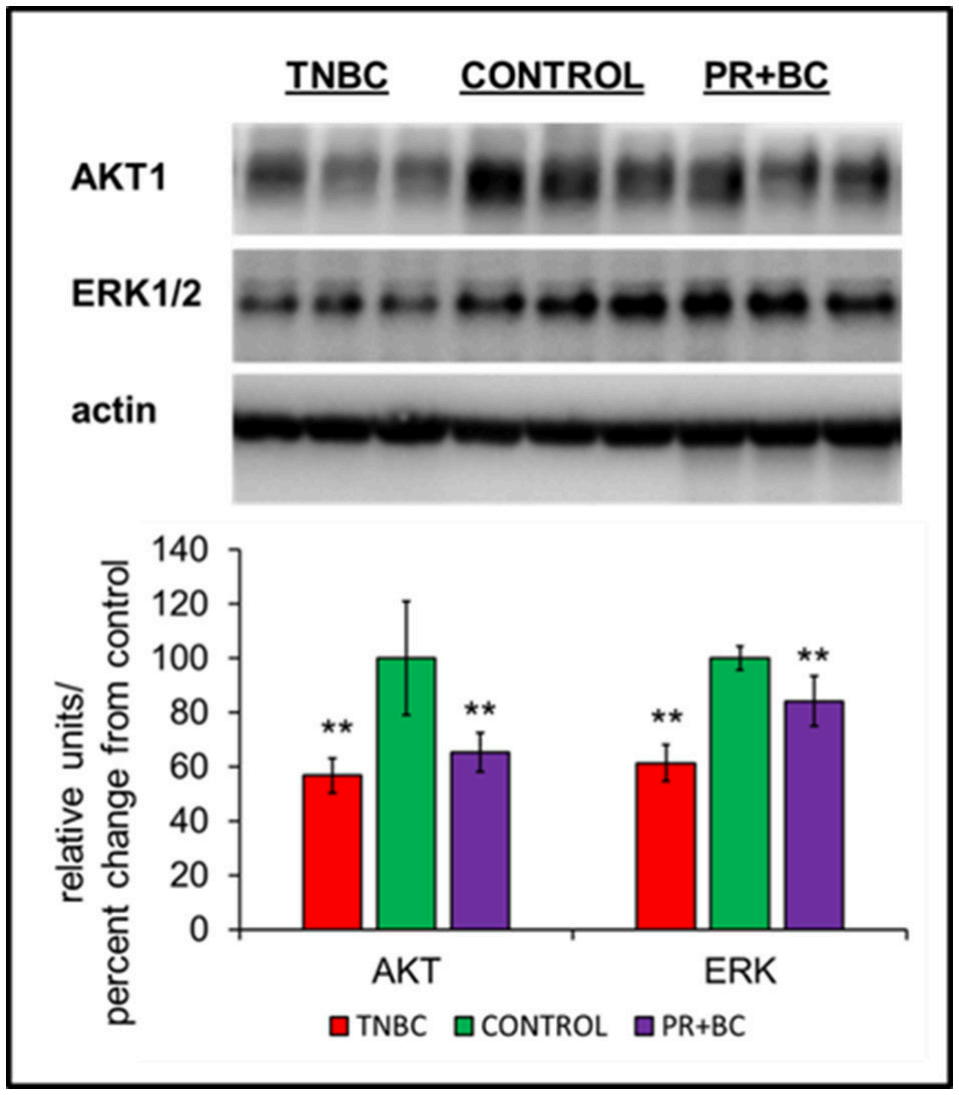
Levels of AKT1 and ERK1/2 in the hippocampal tissues of control and TNBC and PR+BC TumorGraft mice. Data are shown as relative units/percent change from control. Due to protein size differences and scarcity of tissue, membranes were re-used several times. ^**^p < 0.01.

In the ECM-receptor interactions pathways, the GABA A receptor gene was downregulated in the hippocampal tissues of PR+BC animals, and upregulated in TNBC animals. The expression of this gene was further analyzed at the protein level, whereby GABA A Receptor protein was downregulated in both animal groups (Figure 3).

**Figure 4:**
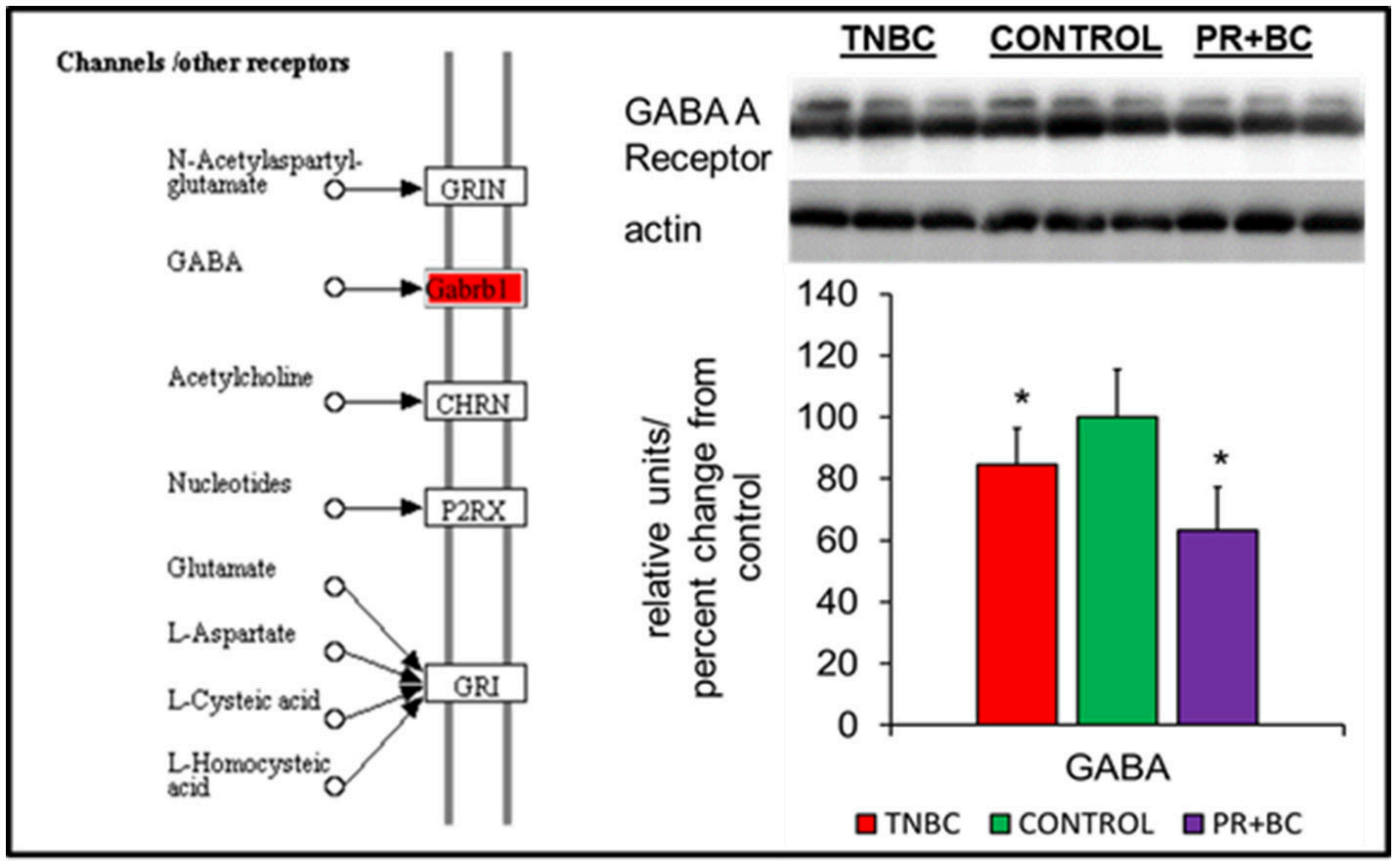
Levels of GABA A Receptor in the hippocampal tissues of control and TNBC and PR+BC TumorGraft mice. The diagram shows the Channels/Other Receptors pathways commonly altered in both TNBC and PR+BC animals, as compared to controls. The graph represents western immunoblotting results. Data are shown as relative units/percent change from control. Due to protein size differences and scarcity of tissue, membranes were re-used several times. ^*^p < 0.05.

Another altered pathway included PI3K-AKT signaling. Protein analysis further revealed statistically significant downregulation of AKT1 and ERK1/2 in the hippocampal tissues of TNBC and PR+BC animals (Figure 4).

### Effect of non-CNS tumor growth on the expression of neuronal PAS domain protein 4 (NPAS) and its targets

**Figure 5:**
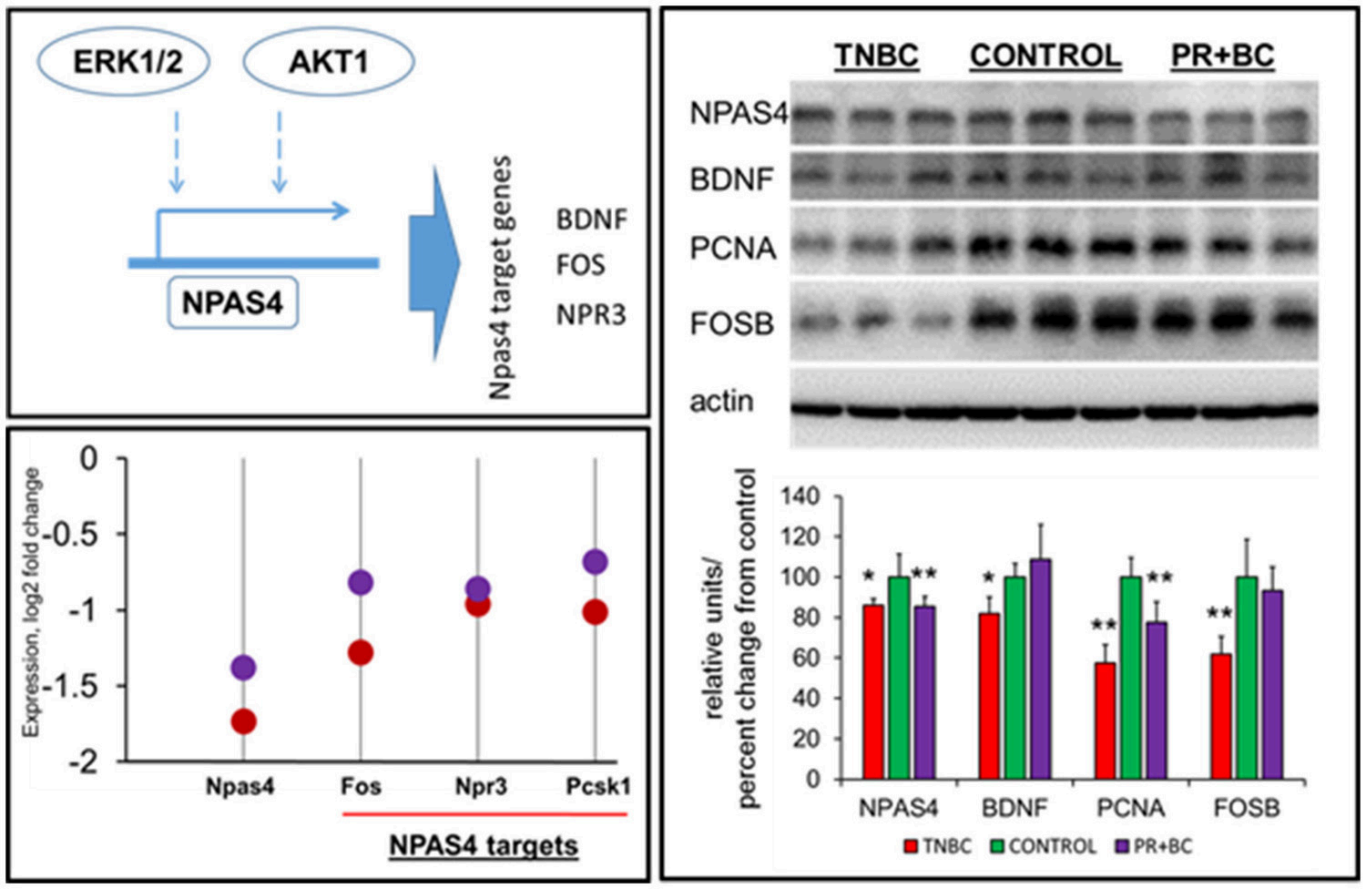
Levels of NPAS4, NPAS4 targets and PCNA in the hippocampal tissues of control and TNBC and PR+BC TumorGraft mice. Schematic representation of the control of NPAS4 protein by AKT1 and ERK1/2 pathways. Expression levels of Npas4 gene and its target genes 9Fos, Npr3, and Pcsk1 in TNBC (red) and PR+BC (purple) animals are given in log2 fold change, as compared to controls. Western immunoblotting was performed to deternime the levels of NPAS4, BDNF, FOSB, and PCNA. The graph represents Western immunoblotting results. Data are shown as relative units/percent change from control. Due to protein size differences and scarcity of tissue, membranes were re-used several times. ^*^p < 0.05; ^**^p < 0.01.

ERK1/2 signaling was reported to regulate NPAS4, which is an important transcription factor. In-depth gene expression analysis revealed that Npas4 was one of the most downregulated genes in the hippocampi of both TNBC and PR+BC tumor-bearing animals, as compared to controls (log fold −1.74 and −1.38, respectively). Along with Npas4, several Npas4 target genes were downregulated, including natriuretic peptide receptor 3 (Npr3), proprotein convertase subtilisin/kexin type 1 (Pcsk1), and FBJ osteosarcoma oncogene (Fos) genes (Figure 5). Further analysis revealed that NPAS4 protein levels were also significantly downregulated in the hippocampi of tumor-bearing mice. Additionally, levels of NPAS4 target proteins—brain-derived neurotrophic factor (BDNF) and FBJ murine osteosarcoma viral oncogene homolog B (FOS B)—were significantly downregulated in the hippocampi of TNBC animals, but not PR+BC animals. Levels of proliferating cell nuclear antigen (PCNA) protein were also downregulated in both TNBC and PR+BC animals (Figure 5).

## Discussion

This study is the first in-depth analysis of the molecular mechanisms of TNBC and PR+BC growth-induced tumor brain manifestations in the hippocampal tissues of TumorGraft mice. The growth of malignant non-CNS tumors had a profound impact on molecular processes in the murine hippocampus. The major findings of our study are as follows: (i) the growth of TNBC and PR+BC tumors significantly altered gene expression in the murine hippocampus; (ii) TNBC tumor growth caused oxidative stress that manifested as significantly elevated levels of 4-HNE; (iii) tumor growth negatively affected the levels of neuronal transcription regulator NPAS4 and its target genes, among them, one of the members of the neurotrophin family of growth factors—BDNF; (iv) tumor growth was associated with significant downregulation of PCNA, AKT 1, and ERK1/2, proteins that are central for the control of neuronal proliferation and survival; and (v) observed molecular changes strongly resembled those associated with neurodegenerative diseases and brain aging. Overall, gene expression changes were more prominent in PR+BC mice than in TNBC mice.

We discovered that TNBC growth causes upregulation of 4-HNE levels in the hippocampus. 4-HNE is a highly reactive, neurotoxic product of lipid peroxidation. Likewise, it is both genotoxic and cytotoxic, and is involved in the pathogenesis of Alzheimer's and Parkinson's diseases, bipolar disorder, and other neurodegenerative and psychiatric diseases (Tsirulnikov et al., 2012; Newton et al., 2017). Increased levels of 4-HNE have been associated with neurodegeneration and Alzheimer's protofibril formation (Siegel et al., 2007). Levels of 4-HNE in the brain are known to be elevated by exposure to ionizing radiation (Mao et al., 2016), alcohol (Tian et al., 2016), low-frequency electric fields (Akpinar et al., 2016), blast injury to the brain (Du et al., 2013), as well as cerebral ischemia (Katayama et al., 2017) and cancer (Zhong and Yin, 2015). Overall, elevated levels of 4-HNE are a sign of brain aging (Benedetti et al., 2014) and neurodegeneration (Farooqui and Horrocks, 2006). In addition, we found a downregulation of ERK1/2 in the hippocampi of tumor-bearing mice. Given recent reports showing the effects of 4-HNE on the levels of MAPK activity in lung cancer cells, as well as the effects of 4-HNE on AKT-mediated regulation of proliferation and apoptosis, analyzing the interplay between MAPK signaling and 4-HNE is of interest.

Another important finding of this study is the downregulation of the neuronal PAS domain protein 4 (Npas4) gene in the hippocampal tissues of TNBC and PR+BC mice, as compared to controls. NPAS4 is a neuron-specific transcription factor that is involved in synaptic plasticity and provides an important link between neuronal activity and memory (Klaric et al., 2017). NPAS4 is important in long-term memory formation in multiple regions of the brain, including the hippocampus, and Npas4 knockout mice fail to form contextual fear memories (Ramamoorthi et al., 2011). The gene may also be implicated in neural circuit plasticity (Sun and Lin, 2016). Moreover, recent studies suggest that NPAS4 may exert neuroprotective effects in ischemic stroke via regulation of cell death and of the inflammatory response (Choy et al., 2015).

**Figure 6:**
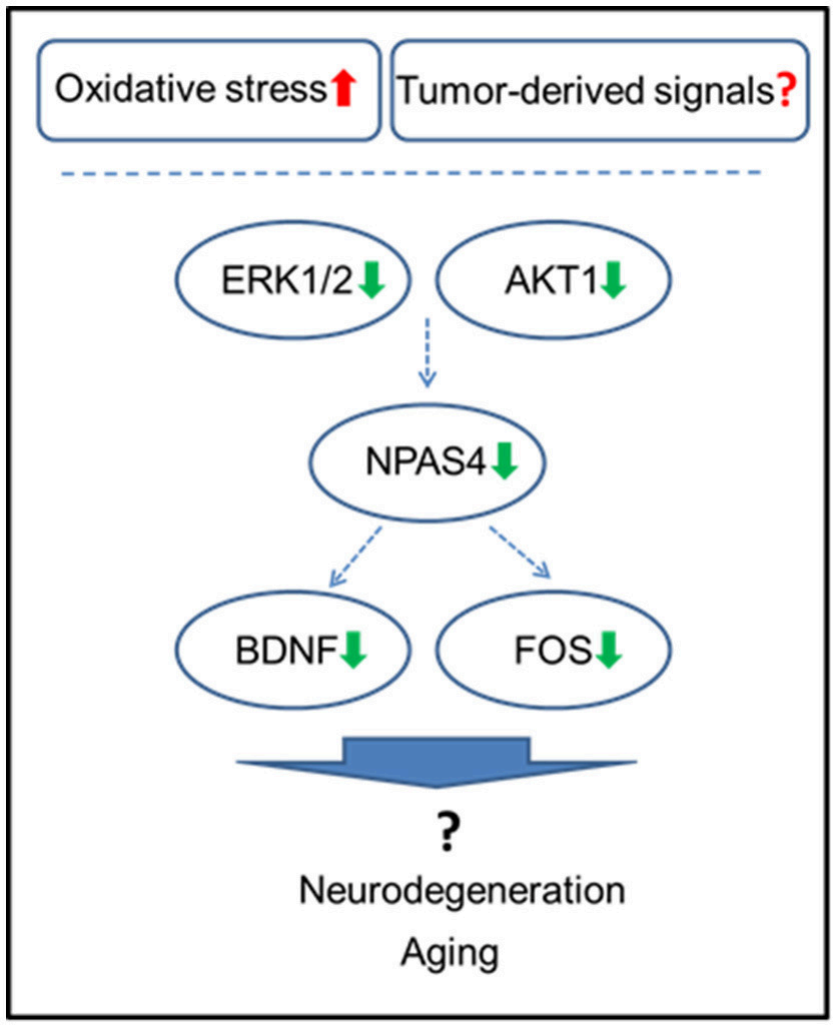
Schematic representation of the roles of NPAS4 and its regulatory network in tumor brain manifestation in the hippocampus.

NPAS4 modulates activity-dependent synaptic connections in both GABAergic and glutamatergic synapses by regulating numerous downstream genes (Sun and Lin, 2016). Among those, NPAS4 has been shown to regulate Bdnf, Nrp3, Fos, and many other genes (Bloodgood et al., 2013; Maya-Vetencourt, 2013). Of those, one of the main NPAS4 targets is BDNF, which belongs to the neurotrophin family and governs and facilitates neuronal differentiation, maturation, growth and survival, and plays a role in neural plasticity (Takami et al., 2005; Park and Poo, 2013). BDNF protein levels were shown to be downregulated by various stressors (Lee and Kim, 2010) and by adverse prenatal environments (Kundakovic and Jaric, 2017). Decreased BDNF levels were reported in Alzheimer's disease, neurodegenerative and psychiatric disorders, stroke, and brain aging, just to name a few (Lee et al., 2005; Park and Poo, 2013) (Figure 6).

NPAS4 also regulates c-Fos and FosB, immediate early response genes that govern cell proliferation and differentiation in response to extracellular stimuli. c-FOS is a marker of neuronal activity, and levels of c-FOS are reduced during brain aging and are associated with age-related decreases in neuronal function and plasticity in various brain regions. c-Fos knockout mice exhibit significant learning impairment and hyperactivity (Velazquez et al., 2015). Meanwhile, downregulated FosB levels have been reported in the hippocampi of both depressed and addicted individuals (Gajewski et al., 2016).

We noted a significant downregulation of Npas4 and its target genes in the hippocampal tissues of TNBC- and PR+BC tumor-bearing animals, as compared to controls. This downregulation of neurotrophins and pro-survival genes may lead to alterations in synaptic plasticity, neuronal survival and maturation, and may in turn underlie memory impairments associated with tumor brain. In the future, it will be important to understand region and cell-type specificity of the observed changes, as well as the time frame for downregulation. The mechanisms of NPAS4 downregulation also need to be analyzed in greater detail. These may be epigenetically regulated, and may involve altered methylation of the NPAS4 promoter (Furukawa-Hibi et al., 2015), or the function of small non-coding RNAs.

Mitogen-activated protein kinase (MAPK) and phosphatidylinositol-3 kinase (PI3K) pathways were shown to partake in the induction of Npas4 expression (Sun and Lin, 2016). Here, we observed downregulation of ERK1/2 and AKT 1, important constituents of these pro-survival pathways. The observed concomitant downregulation of AKT1, ERK1/2, NPAS4, BDNF, and other NPAS4 targets strongly suggests the importance of these pathways in tumor brain, as it manifests in the hippocampus. AKT1 is one of the key cellular oncogenes and its activation is also thought to drive the early steps of cancer, albeit, in the brain, AKT is pivotal for neuronal survival (Datta et al., 1997; Dudek et al., 1997; Akbar et al., 2005). The current study open news avenues to further dissect the potential driving mechanisms and functional consequences of the reduced levels of AKT1, ERK1/2, NPAS4 and BDNF in tumor brain, especially within the concept of aging.

Further studies are needed to investigate the other components of these pathways, and the roles of upstream and downstream signaling. In addition, the inter-relationship between the gene expression and protein levels in the tumor and in the brain tissues need to be further established.

Likewise, it will also be important to discern the mechanisms of transcriptional regulation in tumor brain, as well as the roles of epigenetic changes—such as those in DNA methylation, histone modifications, and small RNAs—in tumor brain's manifestation in the hippocampus, as well as the correlation between the levels of gene expression and protein levels. The timing and interplay between various levels of regulation of gene expression may lead to a better understanding of the initial series of events underlying tumor brain, allowing for the development of novel strategies for diagnosis, prevention, and mitigation.

The hippocampus has been reported as one of the targets of chemo brain. Furthermore, reports of memory impairment in cancer patients prior to diagnosis strongly suggest hippocampal involvement. All of the observed molecular changes—such as increased levels of 4-HNE and decreased levels of AKT1, ERK1/2, NPAS4, PCNA, and BNDF—have negative effects, especially since all of these proteins partake in the regulation of neuronal differentiation and survival. Oxidative stress and the loss of expression of important regulators of neuronal survival and functioning proteins may underlie tumor brain. Some of the TNBC and PR+BC growth-induced changes in the hippocampus were similar to those previously observed in the prefrontal cortex (PFC) of these animals, whereby we noted increased oxidative stress and decreased levels of BDNF. Hence, those mechanisms may be pivotal for tumor brain manifestations in various brain regions. In the future, research should expand to analyze tumor brain in other brain regions.

The analysis of gene expression changes in the hippocampi of TNBC and PR+BC tumor-bearing animals revealed changes in many metabolic pathways, such as galactose metabolism, arachidonic acid metabolism, alpha linolenic acid metabolism, amino acid and sugar metabolism, fatty acid biosynthesis, and many others. This suggests that metabolic alterations are taking place in the hippocampi. These findings may warrant future metabolomics analysis of tumor brain.

In this study, we analyzed tumor brain in animals with breast cancer patient-derived tumor xenografts. In future research, we will seek to expand this analysis and dissect the changes induced by other types of tumors, as well as evaluate tumor-induced brain changes as a function of tumor stage and grade.

Furthermore, molecular changes caused by malignant non-CNS tumor growth on the brain need to be analyzed over the course of tumor development, and to be correlated with the neuroanatomical, cognitive, and behavioral manifestations of tumor brain.

The mechanisms of tumor brain signaling remain obscure, but these signals may be transmitted through the blood. Therefore, it is important to analyze and compare changes in the brain and blood. This will allow us to establish the nature of the tumor brain signal and propose novel blood-based tumor brain diagnostic and prognostic biomarkers. In addition, this study laid foundation for the future large-scale bioinformatic study aimed to establish the links between tumor brain, neurogeneneration and aging.

## Materials and methods

### Animal model

To analyze the effects of non-CNS tumor growth on the brain, we used TumorGraft technology developed by precision medicine company Champions Oncology, Inc. (Baltimore, MD), who provided frozen brain tissues of TumorGraft mice carrying TNBC and PR+BC patient-derived xenografts (PDX). Patients diagnosed with TNBC and PR+BC had their tumors surgically removed and engrafted into mice to generate personalized TumorGraft mouse models for the development of precision oncology applications. All patients provided their informed consent for the use of tumor material for research purposes. The TumorGraft models were generated as previously described (Bertotti et al., 2011; DeRose et al., 2011; Hidalgo et al., 2011; Morelli et al., 2012; Stebbing et al., 2014). Tumor samples were obtained during surgery, and small tumor fragments containing both malignant cells and supportive stromal components were subcutaneously implanted into the flanks of 6-week-old immunodeficient female mice (female *nu*/*nu* athymic mice; Harlan Laboratories, Indianapolis, IND). Animal experiments were approved by Institutional Animal Care and Use Committee protocols. Tumor dimensions were regularly measured and tumor volumes were calculated as previously described (Stebbing et al., 2014). Upon propagation, when TumorGrafts reached more than 200 mm^3^, the animals were divided into groups of three to five. This study focused on the effects in TNBC (*n* = 4), and PR+BC (*n* = 3) animals. Intact animals of the same strain (no tumor, no treatment, *n* = 3) served as baseline controls.

Upon completion of the treatment animals were euthanized using Euthasol overdose; the brains were removed and immediately frozen in liquid nitrogen and stored in −80°C for molecular analysis. The tissues were split to accommodate RNA and protein analysis.

### Gene expression analysis

Hippocampal tissues of three–four animals per group were used for the analysis of gene expression profiles. RNA was extracted from hippocampal tissues using TRIzol® Reagent (Invitrogen, Carlsbad, CA), further purified using an RNAesy kit (Qiagen), and quantified using Nanodrop2000c (ThermoScientific). Afterwards, RNA integrity and concentration were analyzed using 2100 BioAnalyzer (Agilent). Sequencing libraries were prepared using Illumina's TruSeq RNA library preparation kits, and global gene expression profiles were determined using the Next 500 Illumina deep-sequencing platform at the University of Lethbridge Facility. Statistical comparisons between the control and PDX-bearing groups were performed using the DESeq Bioconductor package (version 1.8.3) and the baySeq Bioconductor package (version 1.10.0). Clustering of the samples was analyzed with multidimensional scaling (MDS) plots, built using the plotMDS function from the edgeR Bioconductor package. MA plots showing the relationship between the average level of expression and the log2 fold change were created for each of the comparisons. The MA-plot is a plot of the distribution of the red/green intensity ratio (“M”), plotted by the average intensity (“A”). Features with a false discovery rate (FDR) < 0.1 (10% false positive rate) were considered differentially expressed between conditions. Gene expression datasets are available upon request.

The functional annotations of differentially expressed genes were performed using DAVID, GO (Gene Ontology) Elite, and GO-TermFinder (Boyle et al., 2004). Pathways were visualized using Pathview/KEGG and DAVID Bioinformatics Resources 6.7/KEGG Pathway platforms (Huang et al., 2007; Huang da et al., 2009a,b).

### Western immunoblotting

Western immunoblotting was carried out as previously described (Silasi et al., 2004; Kovalchuk et al., 2016a,b,c). In brief, hippocampal tissues were sonicated in ice-cold 1% SDS and immediately boiled. Protein concentrations were determined using the Bradford assay (BioRad, Hercules, CA). Equal amounts of protein (10–30 μg) were separated by SDS-PAGE into slab gels of 10–15% polyacrylamide and transferred to polyvinylidene difluoride membranes (Amersham Biosciences, Baie d'Urfé, Quebec). Eight membranes were prepared. The membranes were incubated with primary antibodies against 4-HNE, AKT 1, NPAS4 (1:1,000, Abcam), ERK1/2, FOSB, PCNA (1:1,000, Cell Signaling), and actin (1:2,000, Abcam) overnight at 4°C. Primary antibody binding was detected using horseradish peroxidase-conjugated secondary antibodies and the Enhanced Chemiluminescence Plus System (Amersham Biosciences, Baie d'Urfé, Quebec). Chemiluminescence was detected using a FluorChem HD2 camera with FluorChem software (Cell Biosciences). The membranes were stained with Coomassie blue (BioRad, Hercules, CA) to confirm equal protein loading. Signals were quantified using NIH Image J64 software and normalized relative to actin or Coomassie staining.

#### Statistical analysis

Statistical analysis (Student's *t*-test) for DNA methylation, oxidative stress and protein levels was carried out using Microsoft Excel.

## Author contributions

AKo planned and designed the study, performed experiments, analysis and interpretation of data, drafted and revised the manuscript. RR-J participated in gene expression and western blotting analysis. YI conducted bioinformatic analysis of NGS data. DS and AKa provided TumorGraft animal models, took part in study design. BK and OK conceived, designed and supervised the study, revised and finalized the manuscript. All authors read and approved the manuscript.

### Conflict of interest statement

The authors declare that the research was conducted in the absence of any commercial or financial relationships that could be construed as a potential conflict of interest.

## Supplementary material

Figure S1 Clustering of groups and differences between transcriptome profiles in the hippocampal tissues of control and TNBC and PR+BC tumor-bearing mice.

Figure S2 Commonly deregulated pathways in n hippocampal tissues of TNBC and PR+BC tumor-bearing mice.
