# Supplementary figures and images for: Growth of Triple Negative and Progesterone Positive Breast Cancer Causes Oxidative Stress and Down-Regulates Neuroprotective Transcription Factor NPAS4 and NPAS4-Regulated Genes in Hippocampal Tissues of TumorGraft Mice—an Aging Connection
Source: Front Genet. 2018 Mar 5;9:58. doi: 10.3389/fgene.2018.00058 (PMC5845109; doi:10.3389/fgene.2018.00058)

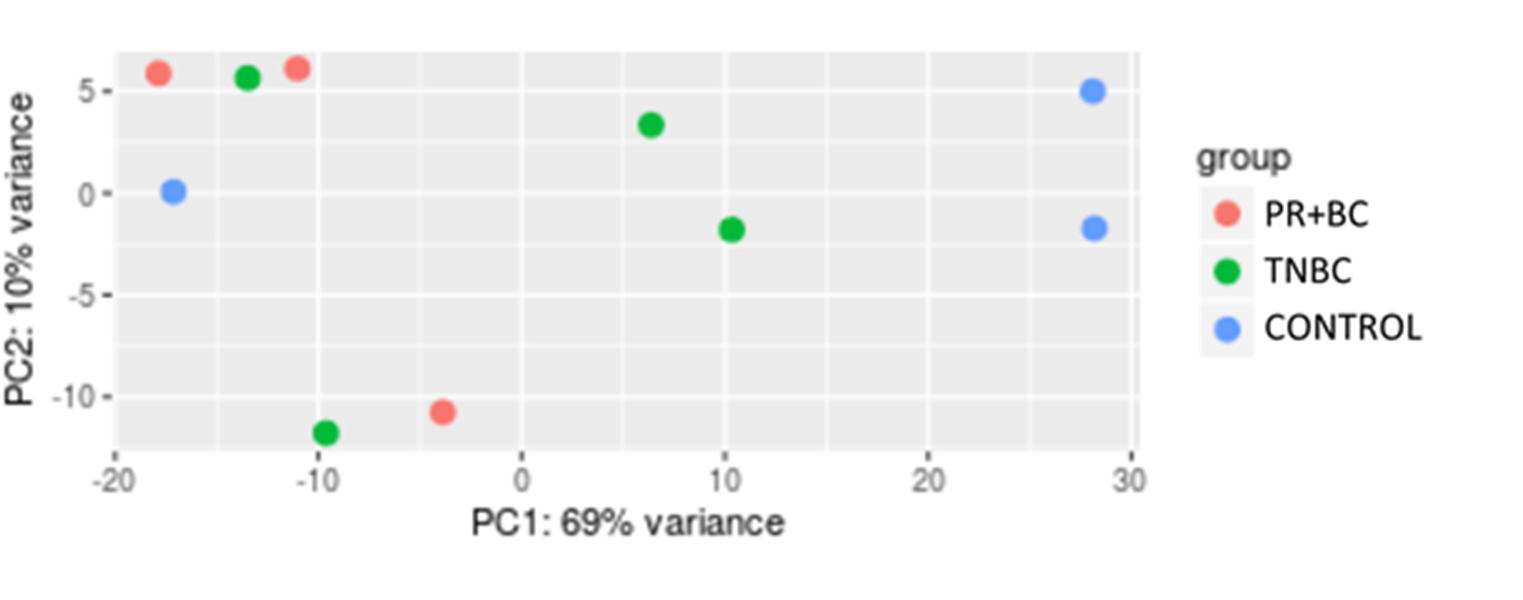

Supplement: Figure S1 — Clustering of groups and differences between transcriptome profiles in the hippocampal tissues of control and TNBC and PR+BC tumor-bearing mice. [file Image1.TIF]

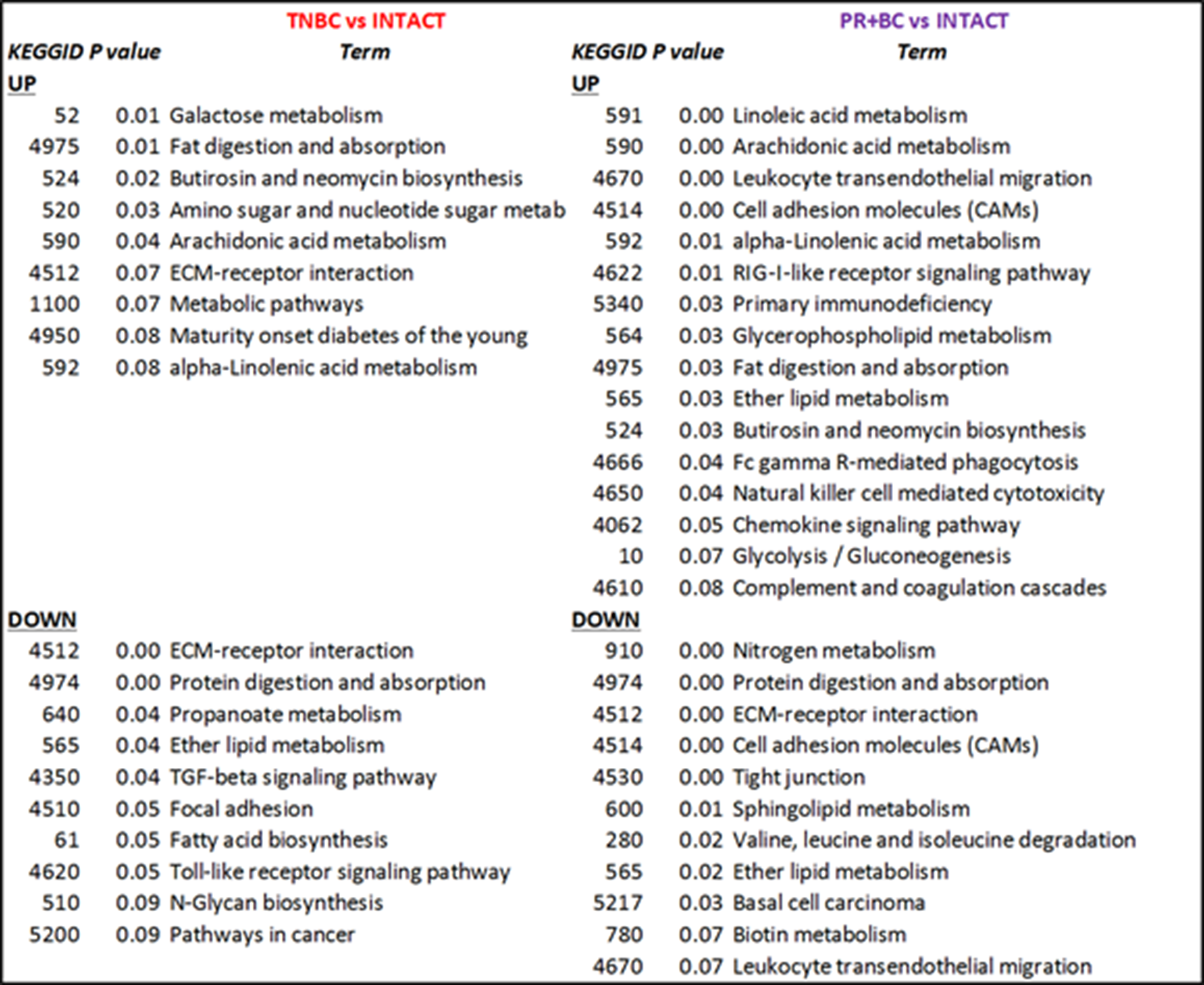

Supplement: Figure S2 — Commonly deregulated pathways in n hippocampal tissues of TNBC and PR+BC tumor-bearing mice. [file Image2.TIF]
